# Minimally invasive vs. traditional tonsillectomy techniques: a systematic review and meta-analysis of randomized controlled trials

**DOI:** 10.3389/fsurg.2026.1802160

**Published:** 2026-06-05

**Authors:** Yanan Zhou, He Ming, Jiao Liang, Qingzhu You

**Affiliations:** 1 Department of ENT, Hospital of Traditional Chinese Medicine of Emeishan City, Leshan, Sichuan, China; 2 College of Traditional Chinese Medicine, Chongqing Three Gorges Medical College, Wanzhou, Chongqing, China

**Keywords:** chronic tonsillitis, efficacy evaluation, meta, minimally invasive, traditional tonsillectomy techniques

## Abstract

**Objective:**

To systematically evaluate both the effectiveness and safety of minimally invasive tonsillar surgical techniques for chronic tonsillitis.

**Methods:**

A comprehensive systematic literature search was performed across four English databases, including PubMed, Cochrane Library, Embase, and Web of Science, to identify randomized controlled trials (RCTs) that exclusively enrolled patients undergoing tonsillectomy only. All included studies compared minimally invasive tonsillectomy with cold dissection tonsillectomy for chronic tonsillitis, with publication dates up to December 25, 2025. Following a rigorous screening process, meta-analyses were carried out using Stata 17.0 and RevMan 5.2.1 software. The study protocol was registered in PROSPERO with registration number CRD420261292929.

**Results:**

A total of 16 randomized controlled trials comprising 1528 patients were included in this study. Compared with cold dissection, minimally invasive surgery tended to improved operative efficiency (MD = −10.48, 95% CI [−13.54, −7.42], *P* < 0.00001, *I*^2^ = 96%) and intraoperative safety (MD = −66.83, 95% CI [−84.35, −49.30], *P* < 0.00001, *I*^2^ = 100%). A series of sensitivity analyses confirmed the robustness of these significant effects. No statistically significant difference was found in early postoperative pain assessed by VAS on day 1 (MD = −0.02, 95% CI [−0.92, 0.87], *P* = 0.96, *I*^2^ = 84%). The incidence of primary postoperative hemorrhage (OR = 0.54, 95% CI [0.17, 1.74], *P* = 0.31, *I*^2^ = 0%) and secondary postoperative hemorrhage (OR = 1.06, 95% CI [0.67, 1.68], *P* = 0.80, *I*^2^ = 0%) did not differ significantly between the two groups. GRADE analysis showed very low certainty for operative time, intraoperative blood loss and VAS, and low certainty for postoperative hemorrhage. All pooled findings need cautious interpretation.

**Conclusion:**

Minimally invasive surgery may be associated with shorter operative time and reduced intraoperative bleeding, but these findings should be interpreted with caution due to unresolved heterogeneity, broad intervention grouping. Further research is needed to confirm these potential benefits and clarify the impact on postoperative hemorrhage.

**Systematic Review Registration:**

https://www.crd.york.ac.uk/PROSPERO, identifier CRD420261292929.

## 1 Introduction

Chronic tonsillitis is a prevalent otorhinolaryngologic disease, particularly common among children and adolescents (1). It is clinically characterized by recurrent acute infections of the palatine tonsils, persistent pharyngeal discomfort, and a sensation of a foreign body in the throat (2, 3). This condition not only leads to frequent medical visits and antibiotic use but may also cause complications such as halitosis, chronic fatigue, and even systemic issues like rheumatic fever or glomerulonephritis, imposing a significant burden on both patients' quality of life and healthcare systems (4–6).

Currently, the management of chronic tonsillitis primarily involves pharmacological therapy and surgical intervention (7). Initial conservative treatment, including antibiotics for acute exacerbations and supportive care, is the mainstay. However, for patients meeting specific criteria, such as frequent recurrent infections (e.g., more than seven episodes in the past year) or complications like peritonsillar abscess, tonsillectomy becomes the definitive and curative surgical intervention. For decades, the conventional “cold” dissection technique, often with electrocautery for haemostasis, has been the standard procedure. While effective in removing the diseased tissue, this method is frequently associated with considerable postoperative pain, delayed return to normal diet and activity, and risks of primary and secondary haemorrhage (8–10).

Driven by the goal of reducing this postoperative morbidity, the field has witnessed the introduction and proliferation of various minimally invasive or “hot” techniques. These technologies, including coblation, radiofrequency, laser (CO_2_ or diode), ultrasonic scalpel, bipolar electrocautery/scissors, and thermal welding, utilize controlled energy to simultaneously cut and coagulate tissue (8–11). Proponents advocate that these methods may offer advantages such as reduced intraoperative blood loss, shorter operative times, and decreased postoperative pain, thereby improving the patient's perioperative experience.

Consequently, a substantial body of randomized controlled trials (RCTs) has been conducted over the past two decades to compare these novel techniques against each other and against cold dissection (8–11) (CD: a technique involving the use of a scalpel, scissors, and snare, without the application of thermal energy. Nevertheless, the evidence remains heterogeneous and often contradictory. Some studies report clear benefits for specific technologies in particular outcomes (8, 12–14), while others find no significant differences or even report disadvantages (12, 15). This inconsistency creates a significant challenge for clinicians seeking to make evidence-based decisions regarding the optimal surgical approach.

Existing systematic reviews and meta-analyses have made valuable contributions but are typically limited to evaluating a single technology or a narrow pairwise comparison, such as coblation vs. all others (11), laser vs. dissection (14, 16), or thermal welding vs. cold dissection (17). While these focused syntheses are informative, they fail to address the broader, pragmatic clinical question: Do minimally invasive tonsillectomy techniques, as a collective category, offer superior perioperative outcomes compared to the established traditional methods?

Therefore, there is a clear need for a comprehensive and updated synthesis that evaluates the aggregate efficacy and safety of the spectrum of minimally invasive techniques vs. traditional dissection. This systematic review and meta-analysis will include all relevant RCTs comparing any recognized minimally invasive tonsillectomy technology with conventional techniques. We will focus on critical, patient-centered outcomes: postoperative pain, operative time, intraoperative blood loss, and postoperative hemorrhage rates. This review aims to provide a clearer, more definitive conclusion on the comparative value of minimally invasive tonsillectomy, thereby guiding clinical practice, patient counseling, and future research directions.

## 2 Method

### 2.1 Search strategy

This systematic review was conducted and reported in accordance with the Preferred Reporting Items for Systematic Reviews and Meta-Analyses (PRISMA) 2020 guidelines. We systematically searched PubMed, Cochrane Library, Web of Science, and Embase databases from their inception to December 25, 2025. The search strategy combined subject headings and free-text terms: MeSH terms were adopted for PubMed and Cochrane Library, while Emtree terms were applied for Embase, alongside corresponding free words.

The search was constructed around three core concepts:Disease: chronic tonsillitis, recurrent tonsillitis, tonsillitis, tonsillar hyperplasia; Interventions: cold dissection, and minimally invasive techniques including radiofrequency, coblation, laser, ultrasound, and microdebrider tonsillectomy; Study Design: randomized controlled trial.

Additionally, we did not search clinical trial registries or grey literature in this study. To minimize the risk of literature omission, we performed manual backward citation screening by reviewing the reference lists of all included studies to retrieve additional eligible publications. The complete detailed search strategies for each database are presented in Supplementary 1 Data Sheet.

### 2.2 Inclusion criteria

Participants (P): Patients with a clinical diagnosis of chronic tonsillitis undergoing isolated tonsillectomy, with no restrictions on age or sex were enrolled. Those diagnosed with tonsillar hypertrophy or obstructive sleep disordered breathing who received concurrent surgeries such as adenoidectomy were excluded.

Intervention/Comparison (I/C): Trials must directly compare minimally invasive tonsillectomy with traditional tonsillectomy by CD. Minimally invasive techniques include, but are not limited to: monopolar/bipolar electrocautery, coblation, radiofrequency, CO_2_ laser, and ultrasonic scalpel.

Outcomes (O): Studies must report at least one of the following primary or secondary outcomes. Primary outcomes: Postoperative pain, operative time, intraoperative bleeding, and postoperative hemorrhage. Secondary outcomes: Time to return to normal diet/activity, overall patient satisfaction, and others.

Study Design (S): Only RCTs were included, where the individual patient was the unit of randomization. We excluded trials where individual tonsils (sides) were randomized, as well as quasi-randomized trials.

### 2.3 Exclusion criteria

Studies meeting any of the following criteria were excluded:

Ineligible Participants: Studies that included patients with acute tonsillitis, infectious mononucleosis, or other primary pharyngeal conditions (e.g., tumors).

Unclear Interventions: Studies with ambiguous surgical technique descriptions (e.g., only mentioning “tonsillectomy” without specifying the method) or insufficient procedural details.

Ineligible Study Design: Non-randomized controlled trials, including observational studies (cohort studies, case-control studies), case series, case reports, reviews, systematic reviews, meta-analyses, conference abstracts, commentaries, and basic science research.

Publication Issues: Duplicate publications, studies for which the full text was unavailable, and studies published in languages other than English.

### 2.4 Data collection

Two researchers (YNZ and HM) independently conducted the literature screening, which followed a two-step procedure. First initial screening: Titles and abstracts were reviewed to exclude obviously irrelevant publication types, such as reviews, animal studies, case reports, and non-randomized controlled trials. Second full-text review: The full texts of potentially eligible studies identified in the initial screening were retrieved and thoroughly assessed to determine final inclusion. Any disagreements arising at any stage of the screening process were resolved through discussion with a third reviewer (QZY) until consensus was reached.

Data Extraction: Upon finalizing the included studies, the following information was systematically extracted to ensure the completeness and comparability of data required for analysis (Table 1).

**Table 1:** Details of data extraction.

Category	Specific details
Basic study information	First author, publication year,country.
Study characteristics	Sample size, gender, age, anesthesia method, follow-up duration and rate, outcomes, ethical, compared tonsillectomy techniques.
Outcome measures	Operative time, intraoperative bleeding, pain score, postoperative hemorrhage and Other outcome information as reported in the studies.

### 2.5 Quality assessment

The methodological quality of the included studies was rigorously appraised in accordance with the guidelines set forth in the Cochrane Handbook for Systematic Reviews of Interventions (18). The appraisal focused on assessing the risk of bias across seven key domains common to randomized controlled trials: random sequence generation, allocation concealment, blinding of participants and personnel, blinding of outcome assessment, completeness of outcome data, selective reporting, and other potential sources of bias.

For each included study, every domain was independently judged as having a “low risk,” “high risk,” or “unclear risk” of bias. Upon completion of all assessments, the overall methodological quality was summarized and visualized in a risk of bias graph. To ensure objectivity and consistency, two reviewers (YNZ and HM) performed this evaluation independently, followed by a cross-verification of their judgments. Any discrepancies were resolved through discussion with a third reviewer (QZY) until a consensus was reached.

In addition, funnel plots, Egger's test, and the trim-and-fill method were used to assess the potential publication bias among the included studies. We assessed the certainty of evidence for each outcome using the GRADE approach (Grading of Recommendations Assessment, Development and Evaluation). The evidence was initially rated as high for randomized trials and low for observational studies, then downgraded based on risk of bias, inconsistency, imprecision, indirectness, and publication bias.

### 2.6 Statistical analysis

This study performed a meta-analysis using RevMan 5.2 and Stata 17.0 software to systematically compare the effects of different surgical techniques. The core outcome measures included operative time, intraoperative bleeding, VAS score, and postoperative hemorrhage (both primary and secondary hemorrhage). Forest plots were generated for each outcome to visually present the effect sizes.

When pooling effect sizes, heterogeneity among studies was first assessed using the *I*^2^ value. When *I*^2^ > 50% was considered indicative of substantial heterogeneity using random effects model; otherwise, a fixed effects model was used. All pooled effect estimates were reported with 95% CI, and a *P* < 0.05 was regarded as statistically significant.

When substantial heterogeneity was detected, we performed meta-regression to examine potential sources, including the specific intervention used in the minimally invasive group, patient population (adults vs. children), and continent. For sensitivity analysis, we sequentially excluded (1) studies with data converted from medians, (2) studies that pooled data across multiple techniques, and (3) influential studies identified through leave-one-out analysis. Subgroup analyses were conducted for age and continent where appropriate.

## 3 Results

### 3.1 Search results

An initial systematic search of English language databases identified 903 potentially relevant records. Following the removal of 315 duplicates using EndNote X7 software, 588 unique records proceeded to the screening stage. Two researchers independently reviewed the titles and abstracts of these records, leading to the exclusion of 466 publications that did not meet the preliminary criteria. The reasons for exclusion at this stage were as follows: 27 study protocols, 43 review articles, 138 studies of other diseases, 3 withdrawn articles, 7 comments, and 248 not meeting the inclusion criteria.

**Figure 1:**
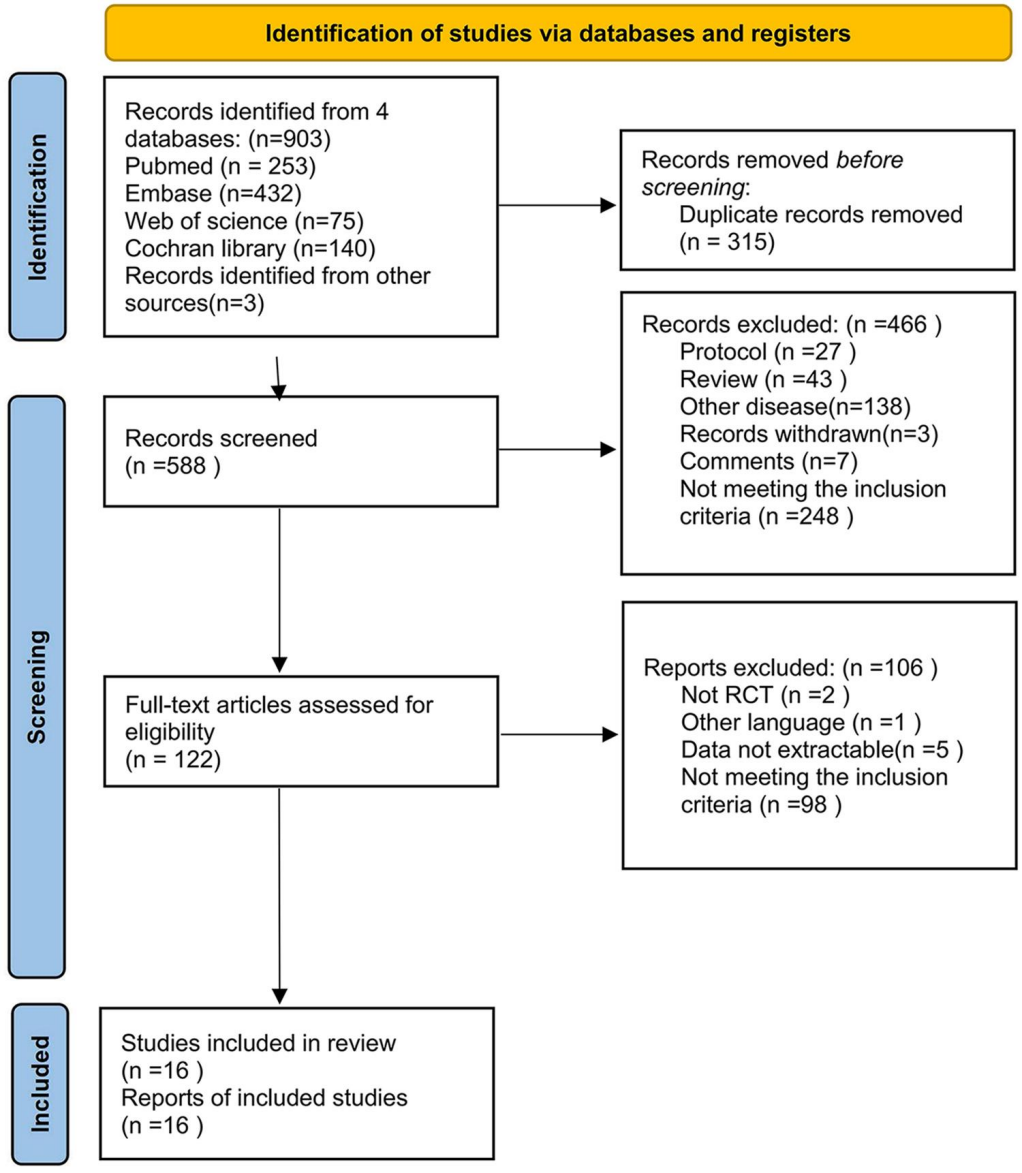
Flow diagram of literature screening and selection outcomes.

Subsequently, full-text assessments were conducted on the remaining 122 articles. During this phase, further exclusions were made: 2 not RCTs, 1 non-English language, 5 data not extractable, and 98 studies of other diseases not meeting the inclusion criteria. Consequently, 16 clinical studies that fulfilled all eligibility criteria were included in the final meta-analysis. The detailed literature selection process is depicted in Figure 1.

### 3.2 Characteristics of the included studies

Based on the inclusion and exclusion criteria, this review ultimately included 16 RCTs published between 2001 and 2024. These studies involved a total of 1,528 patients with chronic tonsillitis who underwent surgical treatment. Among them, 792 patients were assigned to various minimally-invasive technique groups (experiment groups) and 736 patients received CD (control groups). In terms of study design, 13 trials (9, 11, 13, 17, 19–25) were two-arm parallel-group studies, 2 (14, 26) were three-arm parallel-group studies, and 1 (27) was a multi-arm parallel-group study.

#### 3.2.1 Geographic distribution and ethical information

The included studies were geographically diverse, with the majority conducted in Asia 10 studies: Japan 1 (20), Turkey 3 (14, 17, 26), China 1 (12), Iraq 1 (24), India 2 (16, 27), Pakistan 2 (15, 25). 5 studies were from Europe: the United Kingdom 4 (11, 19, 21, 22) and Greece 1 (23). 1 study (14) originated from Egypt in Africa. Additionally, 4 studies (12, 15, 24, 27) did not report obtaining approval from an ethics committee.

#### 3.2.2 Age distribution of patients

Regarding patient age, 10 studies (14–17, 21, 22, 24–27) enrolled only individuals under 18 years old, 4 studies (11, 13, 20, 23) focused on adult populations, and 2 studies (12, 19) included both adult and pediatric participants.

#### 3.2.3 Interventions and outcome measures

In all studies, the control group underwent traditional tonsillectomy, while the experimental group received various minimally-invasive techniques (with slight variations in terminology across studies). 14 studies reported follow-up duration, and 8 of them specified follow-up rates. A total of 23 outcome categories were extracted, with the four most frequently reported being: operative time, intraoperative blood loss, postoperative pain scores, and postoperative hemorrhage (including both primary and secondary bleeding). The basic characteristics of the included studies are detailed in Tables 2, 3.

**Table 2:** Basic characteristics of included literature.

Author year	Country	Sample size		Male/female		Age (years)		Anesthesia		Follow-up duration and follow-up rate	Outcomes	Techniques	Ethical	Surgical method
		E	C	E	C	E	C	E	C					
Raut et al. (19)	UK	100	100	1:2.6		10–54 average 22		NA		15d, 91.5%	①②③④⑤	BS vs. CD	Y	NA
Sugiura et al. (20)	Japan	15	15	NA		21–40		general		6d, NA	②③⑥	UT vs. BT	Y	extracapsular
Raut et al. (21)	UK	18	32	16/34		10–16 average 14.3		NA		17d, NA	①②③④⑤	BS vs. CD	Y	NA
Oko et al. (22)	UK	61	61	NA		5–13	5–13	general		9d, 76.2%	②③④⑦⑧⑨	US vs. BD	Y	NA
						8.4 ± 2.551	8 ± 2.513							
Philpott et al. (11)	UK	43	49	23/69		18–45		general		14d, 77.17%	③④⑩⑪ ⑫⑬⑭	Coblation vs. CD	Y	NA
Stavroulaki et al. (23)	Greece	16	16	3/13	5/11	27.19 ± 8.28	25.56 ± 5.1	general		10d, 100%	②③④⑪⑮⑯⑰⑱	TWS vs. CD	Y	extracapsular
Sezen et al. (17)	Turkey	25	25	NA		9.64 ± 6.24	8.44 ± 5.47	general		7d, 100%	①②③④⑭⑥⑲⑳	TWS vs. CD	Y	extracapsular
Guo and Kong (12)	China	25	39	28/36		10–54 average 22		general		NA	①②③④	Coblation vs. CD	NA	extracapsular
Mahmut et al. (13)	Turkey	80	40	BCD:18/21	24/16	BCD	18–47 average 30.41	general		10d, NA	①②③④	(BCD + TWS) vs. CD	Y	extracapsular
				TWS:17/24		18–53 average 31.27								
						TWS								
						18–49 average 29.55								
Celebi et al. (16)	Turkey	30	30	14/16	16/14	4–6	3–6	general		6m, 100%	①③⑯⑰⑳㉑㉒㉓	TWS vs. CD	Y	NA
						4.97 ± 0.85	5.0 ± 0.85							
Thangavel et al. (15)	India	63	63	26/37	30/33	7–18	7–18	general		1w, NA	①②③	CO_2_ laser vs. CD	Y	extracapsular
						10.75 ± 3.5	9.89 ± 3.1							
Ali et al. (24)	Pakistan	50	50	32/18	29/21	5–15	5–15	NA		NA	①②③	DL vs. CD	NA	NA
						9.52 ± 2.54	10.18 ± 2.86							
Elbadawey et al. (14)	Iraq	97	97	81/113		3–16		general		10d, NA	①②③	RF vs. CD	NA	NA
Özkiriş et al. (26)	Egypt	80	40	Coblation	19/21	5–15		general		2w, 100%	①②③④⑬	(Coblation + DL) vs. CD	Y	extracapsular
				21/19										
				DL 18/22										
Javaid et al. (25)	Pakistan	55	55	38/17	29/26	5–13	5–13	general		6w, NA	①②	BEC vs. CD	Y	extracapsular
						8.8 ± 2.46	9.13 ± 2.38							
Kondra & Choudary (27)	India	34	24	34/24	RF 7–13 average 11.2	7–13 average 11.2		NA		4w, 100%	①②③④	(RF + BRF + CO_2_ laser) vs. CD	NA	NA
					BRF 8–13 average 10.6									
					CO_2_ 8–13 average 10.5									

UK, United Kingdom; NA, not mentioned; E, minimally invasive group; C, control group (cold dissection); m, month; y, year; w, week; d, day.

Outcomes: ①operative time ②intraoperative bleeding ③postoperative pain ④Postoperative Hemorrhage ⑤Complications ⑥Appetit e⑦Gab time ⑧BPD use ⑨Dietary intake score ⑩Otalgia ⑪Analgesia ⑫Swallowing ⑬Normal diet ⑭Normal activities ⑮day of cessation of significant pain [pain score 7 or more] ⑯Nausea ⑰Vomiting ⑱Wound healing ⑲Number of sutures ⑳Anaesthesia time ㉑Extubation time ㉒Delivery time ㉓Pediatric Anesthesia Emergence Delirium(PAED).

BS: bipolar scissors, CD: cold dissection tonsillectomy, BT: blunt dissection tonsillectomy, UT: ultrasonic tonsillectomies, BD: blunt dissection tonsillectomy, US: ultrasonic scalpel tonsillectomy; TWS: Thermal welding system, RF: Radiofrequency，DL: Diode Laser, BRF:Bipolar Radiofrequency, BEC: Bipolar Electro Cautery.

Among them CD, BT, BD belong to the same traditional blunt/cold dissection tonsillectomy technique; UT and US refer to the same ultrasonic scalpel tonsillectomy technique.

**Table 3:** Basic characteristics of included literature.

Author year	Operative Time, Mean ± SD, (min)	Intraoperative Bleeding, Mean ± SD, mL	Pain Score, Mean (SD)	Postoperative Hemorrhage	Other Outcome Information
Raut et al. (19)	BS: Median 13 (range, 3–55)	BS: Median 5 (range, 0–391)	VAS	primary/reactionary rate 2.1%.	Complications
	CD: Median 20 (range, 6–50)	CD: Median 115 (range, 16–642)	overall mean pain score was 6.9 for each group	secondary hemorrhage rate was 16.9%.	BS: 1 tongue minor burn, 1 thermal burn on the cheek.
	*P* < 0.05	*P* < 0.001	*P* > 0.05	*P* > 0.05	CD: 1 loss of the uvula, 2 mouth ulcers,1 middle ear effusion
Sugiura 2002	NA	UT: 4.6 ± 1.9	VAS	NA	Appetite scores
		BT: 41.9 ± 12.9	UT group presented slightly higher mean VAS pain scores during the 6-day period (except for the D2).		D1-D2: BT had higher VAS appetite scores than UT; D3–36: UT scored higher.
		*P* < 0.0001	D1-D6 *P* > 0.05		D1-D6 *P* > 0.05
Raut et al. (21)	BS: Median 10.5 (range, 5–22)	BS: Median 6 (range, 0–121)	VAS	Primary/reactionary rate was 4% (BS 1, CD 1).	Complications: no major complications
	CD: Median 14.5 (range, 6–45)	CD: Median 86 (range, 16–469)	Overall pain scores were 45.7 (CD group) and 43.1 (BS group)	Secondary hemorrhage rate was 14%.	
	*P* < 0.05	*P* < 0.001	*P* > 0.05	BS: 3/18 (16.6%)	
				CD: 4/32 (12.5%)	
				*P* > 0.05	
Oko et al. (22)	NA	US: 3.0 ± 6.88	Face pain scale	Reactive hemorrhage	BPD used: *P* < 0.0001
		BD: 33.1 ± 31.26	US: 1.8 ± 1.046	1.6% for both group.	US: 6/61 (10%) BD: 59/61 (97%)
		*P* < 0.0001	BD: 1.5 ± 0.994	Secondary hemorrhage	Gag time: *P* < 0.0001
			*P* = 0.0003	US: 8/61 (13%)	US: 16.2 ± 4.41(min)
			BD group of pain scores lower than US group.	BD: 6/61 (10%)	BD: 16.7 ± 3.74(min)
			D1, D3 *P* < 0.001; D5, D7, D9 *P* > 0.05.	*P* > 0.05	Dietary intake score: US scored better than BD.
					D1, D5, D7, D9: *P* < 0.05; D3: *P* > 0.05.
Philpott et al. (11)	NA	NA	VAS	No primary hemorrhage in each group.	No significant differences in pain, otalgia, swallowing and analgesia (all *P* > 0.05), except that CD showed worse swallowing difficulty at 6–8 h.
			No significant differences existed between CD and coblation groups.	Secondary haemorrhage: Coblation 11/35, CD 8/36.	Time to resume normal activities (*P* > 0.05).
			*P* > 0.05	*P* > 0.05	CD return to normal diet fast (*P* < 0.05).
Stavroulaki et al. (23)	NA	TWS: 9.4 ± 5.2	VAS	Primary hemorrhage: CD: 2, TWS:0.	Analgesic use was greater in the CD group: CD: 6.88 ± 5.63(acetaminophen)
		CD: 158.44 ± 30.4	Day of cessation of significant pain: TWS: 2.185 ± 2.73	Secondary haemorrhage: CD 1, TWS 0.	TWS: 4.38 ± 4.29(acetaminophen)
		*P* < 0.0001	CD: 5.437 ± 3.59	*P* > 0.05.	TWS discontinued analgesics earlier: CD: 5.50 ± 3.35(days)
			D1-D4 *P* < 0.05		Postoperative nausea/vomiting was rare.
			D0, D5-D10 *P* > 0.05		Tonsillar Fossa Healing:TWS 3 vs. CD 0 at D10.
					All *P* > 0.0.05
Sezen et al. (17)	TWS: 21.52 ± 8.81	TWS: 17.28 ± 13.35	VAS	No primary or secondary hemorrhage in each group.	Anaesthesia time（min): *P* = 0.001
	CD: 36.44 ± 12.07	CD: 132.40 ± 56.97	TWS group shows lower morning time pain scores in D1, D2 *P* < 0.01, D3-D7 *P* > 0.05.		TWS: 40.68 ± 8.98
	*P* = 0.001	*P* = 0.001	TWS group shows lower evening pain scores scores in D1-D7 *P* > 0.05.		CD: 57.72 ± 14.77
					No. Sutures: *P* = 0.001
					TWS: 0.28 ± 0.46
					CD: 2.48 ± 0.96
					Use of pain medication and Return to normal activities: 5 days, *P* > 0.05.
					Appetite: Poor appetite: TWS:1/25 CD: 12/25
					D1 *P* < 0.01, D2-D5 *P* > 0.05
Guo and Kong (12)	Coblation group is shorter than CD group, with an average of 31.2 (min) vs. 12.0 (min), *P* < 0.05	Coblation group is lesser than CD group, with an average of 8.0 (mL) vs. 38.6 (mL). *P* < 0.05	Coblation yielded shorter pain duration than CD (28.8 h vs. 72.6 h). *P* < 0.05	Primary hemorrhage: 1 in CD group in 4h after operative.	NA
				Secondary haemorrhage: CD 1 at D7, Coblation 2 at D4 and D6.	
Mahmut et al. (13)	BCD: 17.73 ± 4.18	BCD: 9.88 ± 3.98	VAS	Primary haemorrhage: CD 1, BCD 1, TWS:0	NA
	TWS: 18.94 ± 3.87	TWS: 10.13 ± 4.14	BCD: 6.35 ± 1.2,39	Secondary haemorrhage: CD 2 at D8, D13; TWS 1 at D7	
	CD: 35.75 ± 10.15	CD: 38.08 ± 10.83	TWS: 4.7 ± 1.3,41	BCD 2 at D5, D7.	
	*P* < 0.05	*P* < 0.05	CD: 4.5 ± 1.2	*P* > 0.05	
Celebi et al. (16)	TWS: 10.08 ± 3.11	NA	Pain/discomfort score:	NA	Duration of anesthesia (min): *P* = 0.000
	CD: 22.17 ± 5.12		TWS: 0.80 ± 1.06		TWS: 20.00 ± 4.48 CD: 32.19 ± 4.31
	*P* = 0.000		CD: 4.77 ± 1.14		Duration of extubation (min): *P* = 0.514
			*P* = 0.000		TWS: 6.15 ± 2.42 CD: 6.23 ± 2.50
					Duration of delivery (min): *P* = 0.514
					TWS: 6.15 ± 2.42 CD: 6.23 ± 2.50
					Nausea-vomiting score: *P* = 0.317
					TWS: 1.00 ± 0.00 CD: 1.07 ± 0.37
					PAED score: *P* = 0.317
					TWS: 8.33 ± 2.06 CD: 12.33 ± 1.15
Thangavel et al. (15)	CO_2_ laser: 31.2 ± 6.64	CO_2_: 40.8 ± 9.7	VAS: D1,D7 *P* < 0.001	NA	NA
	CD: 53.9 ± 12.5	CD: 80.2 ± 18.3	D1: CO_2_: 5.52 ± 1.1		
	*P* < 0.001	*P* < 0.001	CD: 4.87 ± 1.4		
Ifthikhar et al. (15)	Laser: 25.23 ± 10.95	Laser: 28.4 ± 13.86	VAS: *P* > 0.05	NA	NA
	CD: 36.62 ± 12.36	CD: 112.6 ± 56.26	Laser: 7.93 ± 2.76		
	*P* = 0.001	*P* = 0.001	CD: 8.17 ± 2.43		
Ali et al. (24)	RF: 9 ± 1.3	RF: 10 ± 1.4	Wong Baker (FACES): RF group reported lower pain levels.	Reactive hemorrhage: CD 1, RF 0.	NA
	CD: 16 ± 2.	CD: 80 ± 4.8	*P* < 0.01	Secondary hemorrhage: CD 1, RF 0.	
	*P* < 0.001	*P* < 0.001			
Elbadawey et al. (14)	DL: 15 ± 0.83	DL: 25 ± 0.83	Wong Baker (FACES): D1, D7 *P* < 0.05	Reactive hemorrhage: NA	Coblation and CD resumed normal diet on D4, DL on D5. *P* = 0.34.
	Coblation: 10 ± 0.99	Coblation: 20 ± 0.85	D14 *P* > 0.05	Secondary hemorrhage: Coblation: 1 at D7, CD: 1 at D6.	
	CD: 20 ± 1.0	CD: 30 ± 1.0			
	*P* = 0.001	*P* = 0.01			
Javaid et al. (25)	BEC: 19.76 ± 4.47	BEC: 10.9 ± 2.00	NA	NA	NA
	CD: 24.09 ± 4.90	CD: 22.87 ± 2.37			
	*P* < 0.001	*P* < 0.001			
Kondra & Choudary (27)	RF: 17.83 ± 2.2	RF: 30 ± 6.3	VAS: 24h *P* > 0.05	Reactionary haemorrhage: 0	NA
	BRF: 16.30 ± 1.77	BRF: 16.25 ± 4.8	RF: 7.1 ± 0.8	Secondary hemorrhage: RF 1 at 2w.	
	CO_2_: 16.4 ± 2.11	CO_2_: 12.8 ± 5.2	BRF: 7.3 ± 0.6		
	CD: 18.87 ± 2.15	CD: 105.4 ± 24.31	CO_2_: 6.9 ± 0.7		
	*P* < 0.05	*P* < 0.05	CD: 6.9 ± 0.7		
			1w, 2w, 4w *P* < 0.05		

### 3.3 Quality assessment

**Figure 2:**
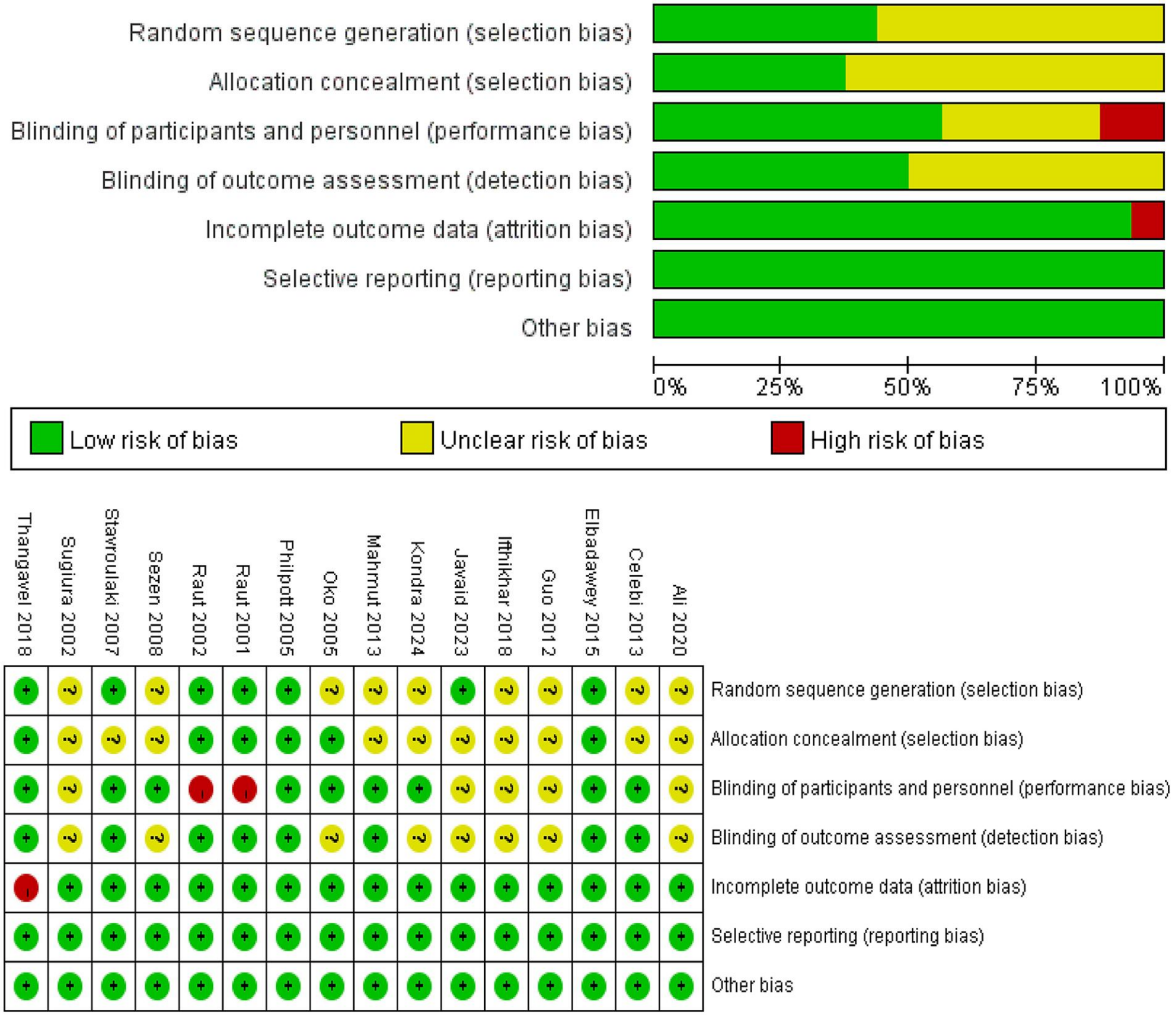
The figure represents the risk of bias assessment for the studies.

In randomization method: adequate random sequence generation was reported in 7 studies, all of which used block randomization (11, 14, 16, 19, 21, 23, 25). The remaining studies only mentioned “random allocation” without specifying the generation method, resulting in a potentially unclear risk of selection bias. In Allocation concealment: 6 studies using a closed envelope system (11, 14, 16, 19, 21, 22). For the remaining studies, the method of allocation concealment was either not implemented or not reported, introducing a potential risk of performance bias. In Blinding Aspects: 6 studies employed a double-blind approach. 2 studies including blinding of patients and nurses (14, 26), 3 studies including patients and outcome assessors (13, 16, 23), and 1 study including patients and the operating surgeon (11). 5 studies using a single-blind method. 3 studies blinding the investigator (19, 21, 22), and 2 studies blinding the patients (17, 27). In Completeness of Outcome Data: Only 1 study (16) reported incomplete data, complete outcome data were available in the remaining studies. The detailed results of the methodological quality assessment are presented in Figure 2.

### 3.4 Meta-analysis

For data extraction and processing, we employed specific conversion and consolidation strategies tailored to the statistical formats reported in different studies, ensuring the feasibility of the meta-analysis and the reliability of its results.

Continuous outcomes (e.g., operative time, intraoperative bleeding): (1) For studies reporting median, range, and sample size like Raut et al. (19); Raut et al. (21), we converted these values to mean and SD using the method recommended by Wan et al. (28). SD ≈ (max − min)/*ξ*(*n*)), with *ξ*(*n*) determined by table lookup; X¯ = (min + 2 × Median + max)/4 + (min − 2 × Median + max)/(4*n*). (2) For the study that reported only the mean without any measure of dispersion like Guo & Kong (12), it was excluded from the meta-analysis. (3) For multi-arm trials as Elbadawey et al. (14), Özkiriş et al. (26); Kondra & Choudary (27), each of which compared multiple minimally invasive groups against a single CD group, we consolidated data from the various minimally invasive arms within each study using the method described by Wan et al. (28) to calculate a weighted mean and pooled SD. Pooled mean = (*n*_1_·*X¯*_1_ + *n*_2_·*X¯*_2_ + … + *n_k_*·*X¯_k_*)/(*n*_1_ + *n*_2_ + … + *n_k_*), where *n* is the sample size of each arm and X¯ is the estimated mean of each arm. Pooled SD = sqrt([(*n*_1_ − 1)*S*_1_^2^ + (*n*_2_ − 1)*S*_2_^2^ + … + (*n_k_* − 1)*S_k_*^2^]/[(*n*_1_ + *n*_2_ + … + *n_k_*) − *k*]), where *S* is the estimated standard deviation of each arm. This combined “minimally invasive” dataset was then compared with the CD group in the meta-analysis.

Dichotomous outcomes (e.g., incidence of primary/secondary hemorrhage): For multi-arm studies reporting dichotomous data for multiple minimally invasive groups, we adopted a direct and conservative approach: the total number of events and the total sample size from all minimally invasive arms within a single study were summed. This pooled event count and total sample size were then used to represent the overall minimally invasive group for that study. The detailed conversion steps and calculations are documented in Supplementary 2 Data Sheet.

#### 3.4.1 Operative time

**Figure 3:**
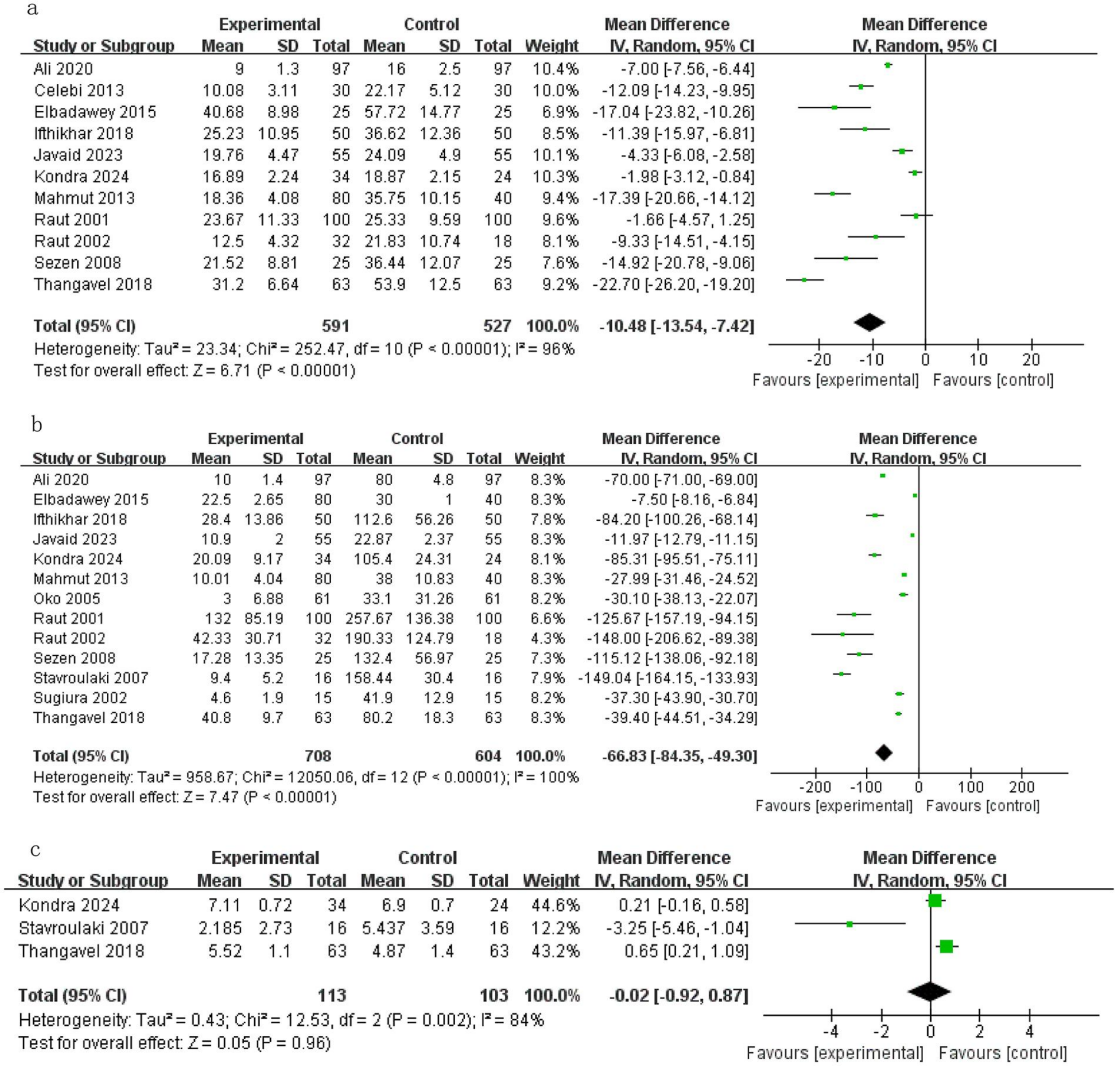
The figure represents a forest plot of operative time **(a)**, forest plot of intraoperative bleeding **(b)**, forest plot of VAS scores **(c)**.

In the comparison of operative time, a total of 12 studies were included. Following the exclusion of 1 study (12), the analysis of 11 studies revealed substantial heterogeneity among the studies (*I*^2^ = 96%, *P* < 0.00001), a random effects model was used. The results showed the operative time in the minimally invasive surgery group was significantly shorter than that in the CD group (MD = −10.48, 95% CI [−13.54, −7.42]). The difference was statistically significant (*P* < 0.00001). This finding suggests that minimally invasive techniques suggesting a potential advantage over traditional methods (CD group) in reducing operative time. Detailed results are shown in Figure 3a. According to the GRADE rating system, the overall certainty of evidence for this outcome was graded as very low. Detailed results are shown in Supplementary 10 Data Sheet.

#### 3.4.2 Intraoperative bleeding

A total of 14 studies were identified reporting on intraoperative bleeding. Following the exclusion of 1 study (12), 13 studies were synthesized finally. The meta analysis revealed considerable heterogeneity among the included studies (*I*^2^ = 100%, *P* < 0.00001), a random effects model was used. The results demonstrated a statistically significant reduction in intraoperative bleeding in the modern minimally invasive surgery group compared to the CD group (MD = −66.83, 95% CI [−84.35, −49.30], *P* < 0.00001). Detailed results are shown in Figure 3b. According to the GRADE rating system, the overall certainty of evidence for this outcome was graded as very low. Detailed results are shown in Supplementary 10 Data Sheet.

#### 3.4.3 VAS

The clinical descriptions of postoperative pain varied across the 14 included studies. 1 study (22) use face pain scale,1 study (13) use pain/discomfort score, 2 studies (14, 24) use FACES and 10 studies employed the VAS to assess postoperative pain. Among them, data from 4 studies (15, 16, 23, 27) were suitable for conversion and subsequent meta-analysis. As the timing of VAS assessments varied across studies, we selected a common time point for comparison. 3 of these studies reported VAS scores on Postoperative Day 1, which were included in the pooled analysis. Considerable heterogeneity was observed among the studies (*I*^2^ = 84%, *P* = 0.002), a random-effects model was applied. The pooled results showed no statistically significant difference in VAS scores between the minimally invasive surgery group and the CD group (MD = −0.02, 95% CI [−0.92,0.87], *P* = 0.96). Detailed results are presented in Figure 3c. According to the GRADE rating system, the overall certainty of evidence for this outcome was graded as very low. Detailed results are shown in Supplementary 10 Data Sheet.

#### 3.4.4 Postoperative hemorrhage

##### 3.4.4.1 Primary hemorrhage

A total of 9 studies (11, 12, 17, 21–24, 26, 27) reported on the incidence of primary/reactionary hemorrhage (bleeding occurring within first 24 h after surgery). 3 of the included studies (11, 23, 27) reported 0 cases of primary hemorrhage. 2 studies (19, 21) adopted conservative treatment, including anti-infection therapy, analgesia, and hydrogen peroxide mouthwash. 2 studies (17, 24) recorded surgical intervention by returning patients to the operating room. Another three studies (12, 22, 26) did not specify the exact hemostatic measures applied. 4 studies (11, 22, 23, 27) showed no significant difference in the incidence of primary hemorrhage between the two groups. 1 study (21) indicated that the primary hemorrhage rate was lower in the CD group than in the minimally invasive group. 4 studies (12, 17, 24, 26) reported that the primary hemorrhage rate was lower in the minimally invasive group than in the CD group.

**Figure 4:**
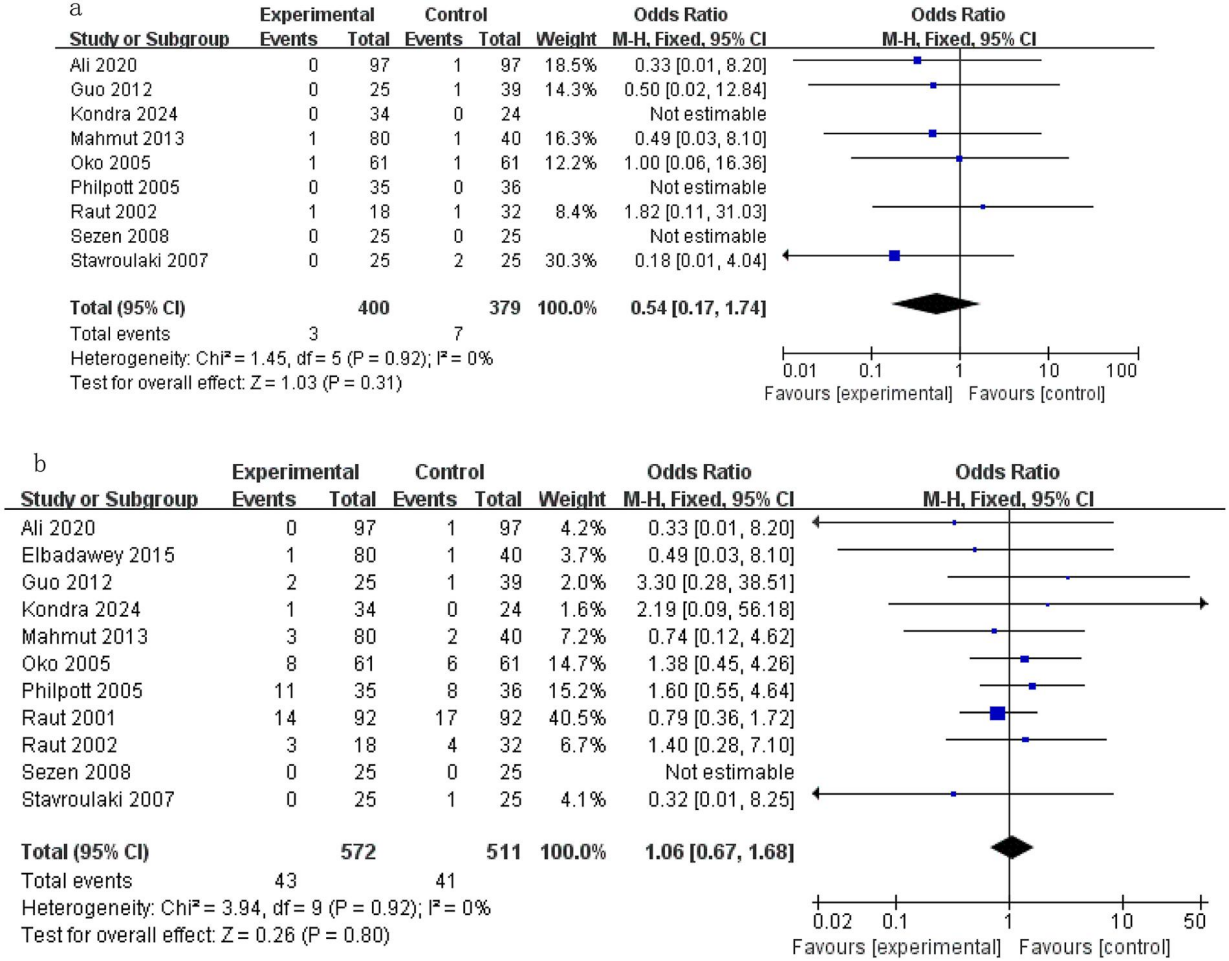
The figure represents a forest plot of primary hemorrhage **(a)**, forest plot of secondary hemorrhage **(b).**

The meta analysis revealed low heterogeneity among the included studies (*I*^2^ = 0%, *P* = 0.92), a fixed effect model was applied. The pooled analysis demonstrated no statistically significant difference in the incidence of primary hemorrhage between the minimally invasive surgery group and the CD group (OR = 0.54, 95% CI [0.17, 1.74], *P* = 0.31). According to the GRADE rating system, the overall certainty of evidence for this outcome was graded as low. Detailed results are shown in Figure 4a, Supplementary 7 and 10 Data Sheet.

##### 3.4.4.1 Secondary hemorrhage

A total of 11 studies (11, 12, 14, 17, 19, 21–24, 26, 27) reported on the incidence of secondary hemorrhage (bleeding occurring more than 24 h postoperatively). 1 study (23) reported no significant difference in the rate of secondary hemorrhage between the two surgical groups. 6 studies (11, 12, 17, 21, 22, 27) found a lower incidence in the CD group compared to the minimally invasive group. Conversely, 4 other studies (14, 19, 24, 26) demonstrated that the incidence of secondary hemorrhage was lower in the minimally invasive group than in the CD group. Regarding secondary hemorrhage, most cases were managed conservatively. Only 3 studies (11, 19, 22) reported that patients with substantial bleeding underwent reoperation.

The meta analysis revealed low heterogeneity among the included studies (*I*^2^ = 0%, *P* = 0.92), and therefore a fixed effect model was applied. The pooled analysis demonstrated no statistically significant difference in the incidence of secondary hemorrhage between the minimally invasive surgery group and the CD group (OR = 1.06, 95% CI [0.67, 1.68], *P* = 0.80). According to the GRADE rating system, the overall certainty of evidence for this outcome was graded as low. Detailed results are shown in Figure 4b, Supplementary 7 and 10 Data Sheet.

#### 3.4.5 Sensitivity analysis, meta-regression and subgroups analysis

##### 3.4.5.1 Operative time

To explore the sources of high heterogeneity in the meta-analysis of operative time, we conducted a series of sensitivity analyses. Firstly, exclude studies with data converted from medians (19, 21), heterogeneity remained high (*I*^2^ = 96.7%, MD = −11.63, 95% CI [−15.07, −8.20], *P* < 0.001). Secondly, excluded studies involving the consolidation of multiple technique group data (14, 26, 27) also yielded significant heterogeneity (*I*^2^ = 95.6%, MD = −11.76, 95% CI [−15.85, −7.67], *P* < 0.001). Thirdly, analysis by sequentially removing individual studies indicated that the heterogeneity was primarily attributable to study Kondra & Choudary (27). However, even after excluding these outliers, a substantial level of heterogeneity persisted**.**

Notably, all sensitivity analyses consistently and robustly demonstrated that minimally invasive surgery significantly reduced operative time, with MD estimates ranging from −11.76 to −10.78. This pattern indicates that the high observed heterogeneity likely stems from underlying clinical or methodological variations across the studies, rather than from the data processing methods employed.

Meta-regression showed no significant overall model effect (*F* = 0.21, *P* = 0.89), with adjusted *R*^2^ = −30.73%, indicating covariates did not explain between-study heterogeneity. All covariates were non-significant (continent: *β* = −0.75, *P* = 0.85; age: *β* = 0.66, *P* = 0.84; method: *β* = 0.66, *P* = 0.47), and residual heterogeneity remained high (*I*^2^_res = 95.65%). The included covariates were not major sources of heterogeneity, suggesting other unmeasured factors may explain the variation.

Subgroup analysis showed that high heterogeneity still existed within each subgroup, suggesting that besides age, other factors such as surgical technique and device parameters may also influence operative time. See Supplementary 3 Data Sheet for details.

##### 3.4.5.2 Intraoperative bleeding

Regarding the high heterogeneity in the Meta-analysis of Intraoperative Bleeding, the methodological approach was consistent with that used for operative time. The initial analysis showed high heterogeneity (*I*^2^ = 100%, MD = −66.83, 95% CI [−84.35, −49.30], *P* < 0.00001). After excluding studies with converted data of Raut 2001 (19) and Raut 2002 (21), heterogeneity remained unchanged (*I*^2^ = 99.9%, MD = −58.55, 95% CI [−77.08, −40.03], *P* < 0.001). A further step excluding studies with pooled data also yielded persistent high heterogeneity (*I*^2^ = 99.9%, MD = −66.28, 95% CI [−94.11, −38.44], *P* < 0.001). Sequential elimination of individual studies indicated that the heterogeneity was primarily attributable to Ali 2020, Javid 2023 and Elbadawey 2015; however, even after their exclusion, the pooled effect size still indicated that the intervention could significantly reduce intraoperative blood loss, with no change in the direction of the conclusion. Across all sensitivity analyses, the conclusion that minimally invasive surgery significantly reduces intraoperative blood loss remained robust, with MD estimates ranging approximately from −78.81 to −58.55.

Meta-regression of continent, age, and minimally invasive method showed no significant overall model effect (*F* = 1.10, *P* = 0.40) and no significant covariates.

Subgroup analysis showed a statistically significant difference between groups (*P* = 0.000), suggesting that the grouping factor may be the main source of heterogeneity. High heterogeneity remained in both the child and adult subgroups, while the mixed-age subgroup showed no heterogeneity (*I*^2^ = 0.0%), although this subgroup had an extremely low weight. Detailed results are shown in Supplementary 4 Data Sheet.

##### 3.4.5.3 VAS

Regarding the high heterogeneity in the Meta-analysis of VAS pain scores, the methodological approach was consistent with that used for operative time. The initial analysis showed high heterogeneity (*I*^2^ = 84%, MD = −0.02, 95% CI [−0.92, 0.87], *P* = 0.96). After excluding studies with pooled data, heterogeneity persisted high (*I*^2^ = 93.6%, MD = −0.77, 95% CI [−3.70, 2.16], *P* < 0.001). Detailed results are shown in Supplementary 5 Data Sheet.

#### 3.4.6 Publication bias

Publication bias was evaluated via funnel plots, Egger's test, and trim-and-fill analysis. For both operative time (*P* = 0.189) and intraoperative bleeding (*P* = 0.334), funnel plots showed asymmetric trends but non-significant Egger's tests. Trim-and-fill analysis required no imputation of studies, with no change in effect sizes, confirming the robustness of our findings. Detailed results are shown in Supplementary 8 and 9 (Data Sheet).

## 4 Discussion

This systematic review and meta-analysis comprehensively evaluated the efficacy and safety of minimally invasive surgery for chronic tonsillitis. The study indicates that minimally invasive surgery demonstrates clear and potential advantage in shortening operative time and reducing intraoperative bleeding; however, these benefits do not extend to postoperative pain relief or the reduction of postoperative hemorrhage risk.

Notably, in the analyses that demonstrated significant benefits in operative time and intraoperative bleeding, we observed very high heterogeneity, prompting a series of deep investigations. The conclusion drawn is that in the surgical treatment of chronic tonsillitis, minimally invasive surgery possesses a trend toward benefit in efficiency and safety compared with the traditional snare technique, but no significant difference was found in postoperative recovery indicators.

In terms of surgical efficiency and intraoperative safety: The pooled analysis showed that minimally invasive surgery significantly shortened operative time (MD = −10.48, 95% CI [−13.54, −7.42], *P* < 0.00001) and markedly reduced intraoperative bleeding (MD = −66.83, 95% CI [−84.35, −49.30], *P* < 0.00001). This benefit can be attributed to the precision and simultaneous hemostatic capability of minimally invasive technologies, such as low-temperature plasma and laser (29, 30).

Although the effect sizes for operative time and intraoperative bleeding were highly significant, both analyses exhibited extremely high heterogeneity (*I*^2^ > 90%). Therefore, we performed systematic sensitivity analyses meta-regression and subgroup to test the robustness of the results and explore sources of heterogeneity.

Regardless of whether specific studies or data-processing methods are excluded, sensitivity analyses consistently show that minimally invasive surgery significantly reduces operative time and intraoperative bleeding, and the conclusions remain stable, unaffected by individual studies.

Meta-regression analysis suggests that the sources of heterogeneity are complex. In this study, patient age distribution, continent of study origin, and surgical method were included as covariates. The results showed that none of these factors significantly explained the heterogeneity. Further subgroup analysis revealed that, for intraoperative bleeding, the mixed-age subgroup showed no heterogeneity, suggesting that age may be a potential factor to consider. Overall, limited by the current number of studies, the high heterogeneity observed in this study may stem from more complex, unmeasured potential factors, such as differences in clinical practice (e.g., specific device parameter settings) and nuances in intraoperative hemostatic strategies.

For postoperative pain, we meta-analyzed data at postoperative day 1 due to varied evaluation methods and time points, finding no significant between-group difference. We thus adopted descriptive analysis to synthesize the current evidence (Supplementary Material 6 in data sheet). Among the 14 included studies comparing postoperative pain between minimally invasive techniques and CD group, 7 studies (12–14, 17, 23, 24, 27) demonstrated that minimally invasive surgery was associated with significantly less pain (*P* < 0.05), mainly in the early postoperative period. In contrast, 3 studies (16, 22, 26) showed that CD was significantly superior in pain control (*P* < 0.05). 2 (11, 19) studies suggested a nonsignificant advantage of minimally invasive surgery. TWS, coblation, and RF were associated with better early pain relief. These findings indicate that the analgesic advantage of minimally invasive techniques is time dependent and technique specific.

Postoperative hemorrhage: The analyses for both primary hemorrhage (OR = 0.54, *P* = 0.92) and secondary hemorrhage (OR = 1.06, *P* = 0.80) showed low heterogeneity and no significant differences. This is an important and reliable safety finding, indicating that, when surgical indications and operative standards are strictly adhered to, minimally invasive surgery does not increase the risk of this major complication, and its safety profile is comparable to that of CD.

Evidence quality was assessed using the GRADE framework. The certainty of evidence for operative time and intraoperative bleeding was rated as very low, primarily due to serious risks of bias and extreme heterogeneity. Similarly, the evidence for the postoperative day 1 VAS pain score was rated as very low, attributed to high risk of bias, substantial heterogeneity, and serious imprecision. For primary and secondary hemorrhage, the certainty was low, owing to biased study designs and insufficient event numbers resulting in wide confidence intervals. Overall, the limited certainty of the evidence restricts reliable interpretation of the pooled results. Large-sample, rigorously designed randomized controlled trials are still needed to confirm the clinical advantages of minimally invasive tonsillectomy. Detailed results are presented in Supplementary 10 Data Sheet.

Based on the above analyses, the main findings were interpreted as follows: (1) Prudent interpretation of therapeutic benefits. Minimally invasive surgery yielded clear, potential advantage in shortening operative time and reducing intraoperative blood loss, improving surgical efficiency and intraoperative visualization. Despite stable results confirmed by sensitivity analysis, cautious clinical interpretation is still warranted given the high heterogeneity. (2) Validation of safety equivalence. Minimally invasive surgery did not increase the risk of postoperative hemorrhage, reassuring clinical safety concerns. Nevertheless, relevant guidelines published in Otolaryngology-Head and Neck Surgery (7) recommend detailed documentation of postoperative bleeding events. At present, most primary studies lack sufficient descriptions of hemorrhage severity, including intervention requirements, blood loss thresholds and specific management measures. This results in ambiguous available evidence and hinders in-depth comparison and pooled analysis. Accordingly, future studies should systematically record hemorrhage grading and clinical management details to generate comparable and high-quality evidence, so as to provide reliable references for surgical selection. (3) Complex causes of non-superiority in pain relief. The pooled analysis showed no difference in postoperative day 1 pain, but this does not definitively rule out a pain advantage for minimally invasive techniques. Substantial heterogeneity in pain assessment across studies, including variations in pain scales, timing, analgesic regimens, and reporting methods, which may obscure the theoretical trauma-reduction advantage of minimally invasive techniques. Postoperative pain is influenced by multiple factors, including varying minimally invasive techniques, inconsistent analgesic regimens, and assessment timing. In future reseach standardized outcome assessment and independent evaluation of each technique are needed to clarify their analgesic profiles.

## 5 Limitation

This study has several limitations. (1) This study has methodological limitations. Several included trials lacked detailed descriptions of randomization, allocation concealment and consistent blinding implementation, which may undermine result validity and generalizability. Primary outcomes were graded as very low-certainty evidence, further weakening conclusion reliability. Future RCTs should standardize blinding design and outcome assessment to minimize bias and enhance result comparability. (2) Unidentified sources of heterogeneity for key outcomes. Although meta-regression and subgroup analyses were performed for further exploration, the substantial heterogeneity in operative time and intraoperative blood loss could not be fully explained. Undescribed differences in surgical method, such as device parameters and operative details, represent important potential confounding sources. Future studies should provide more detailed descriptions of surgical techniques. (3) Substantial methodological heterogeneity in pain assessment. Marked methodological heterogeneity existed in the evaluation of postoperative pain, including inconsistent assessment tools and time points, which directly influenced the pooled analysis and clinical interpretation of the results. (4) Insufficient attention to patient-reported outcomes. Only a small number of included studies focused on patient-reported outcomes such as long-term quality of life (9, 11, 17) and taste function (9), limiting a comprehensive evaluation of the overall benefits of minimally invasive surgery.

To address the above limitations and verify the present findings, well-designed, standardized multicenter randomized controlled trials are needed in the future to provide higher-level clinical evidence.

## 6 Conclusion

For chronic tonsillitis, minimally invasive surgery may be associated with shorter operative time and reduced intraoperative bleeding, without increasing the risk of postoperative hemorrhage. Nevertheless, these findings should be interpreted with caution due to unresolved heterogeneity and broad intervention grouping. Based on the currently available evidence with substantial heterogeneity in pain assessment, the pooled analysis at postoperative day 1 revealed no statistically significant difference between groups. However, this finding should be interpreted with caution due to the variability in pain scales, evaluation time points, analgesic regimens, and reporting methods across studies. In clinical practice, physicians and patients should emphasize on its intraoperative efficiency and safety. Future research should concentrate on standardizing perioperative assessment and reporting, so as to further confirm these potential benefits and clarify the key factors influencing the heterogeneity of treatment outcomes.

## Data Availability

The original contributions presented in the study are included in the article/Supplementary Material, further inquiries can be directed to the corresponding author.
