# Supplementary material for: Minimally invasive vs. traditional tonsillectomy techniques: a systematic review and meta-analysis of randomized controlled trials
Source: Front Surg. 2026 Jun 5;13:1802160. doi: 10.3389/fsurg.2026.1802160 (PMC13279436; doi:10.3389/fsurg.2026.1802160)

Supplementary materials

[Supplement 1. Search strategy 1](#_Toc25579)

[Supplement 2. Different categories data re-integrated for studies 3](#_Toc12908)

[Supplement 3：Sensitivity analysis, meta-regression and subgroup of operative time 6](#_Toc18780)

[Supplement 4：Sensitivity analysis, meta-regression and subgroup of Intraoperative Bleeding 9](#_Toc30435)

[Supplement 5：Sensitivity analysis of VAS 13](#_Toc27191)

[Supplement 6: Pain comparison outcomes 14](#_Toc25524)

[Supplement 7: Hemorrhage outcomes 16](#_Toc26436)

[Supplement 8: Publication bias of operative time 18](#_Toc3372)

[Supplement 9: Publication bias of intraoperative bleeding 19](#_Toc18334)

#

# Supplement 1. Search strategy

| Pubmed | | |
| --- | --- | --- |
| #1 | "Tonsillitis"[Mesh] OR "Tonsillitis/surgery"[Mesh] OR ("chronic tonsillitis"[tiab] OR "recurrent tonsillitis"[tiab] OR "tonsillar hyperplasia"[tiab]) | 9086 |
| #2 | "Tonsillectomy"[Mesh] OR "Ablation Techniques"[Mesh] OR ("tonsillectom*"[tiab] OR "tonsillotom*"[tiab] OR "coblation"[tiab] OR "cold dissection"[tiab] OR "electrocauter*"[tiab] OR "radiofrequency ablation"[tiab] OR "laser tonsillectomy"[tiab] OR "ultrasonic tonsillectomy"[tiab]) | 164959 |
| #3 | "Randomized Controlled Trial" [Publication Type] OR "Randomized Controlled Trials as Topic"[Mesh] OR "randomized"[tiab] OR "randomly"[tiab] OR "placebo"[tiab] OR "clinical trial"[tiab] OR "RCT"[tiab] | 1651625 |
| #4 | #1 AND #2 AND #3 | 253 |
| Web of sciences | | |
| #1 | TS=(“chronic tonsillitis” OR “recurrent tonsillitis” OR “tonsillar hyperplasia”) | 651 |
| #2 | TS=(tonsillectom* OR tonsillotom* OR coblation OR “cold dissection” OR electrocauter* OR “radiofrequency ablation” OR “laser tonsillectomy” OR “ultrasonic tonsillectomy”) | 32449 |
| #3 | TS=(randomized OR randomised OR randomly OR “clinical trial” OR RCT OR placebo) | 1191024 |
| #4 | #1 AND #2 AND #3 | 75 |
| Embase |  |  |
| #1 | 'tonsillitis' OR 'chronic tonsillitis':ti,ab,kw OR 'recurrent tonsillitis':ti,ab,kw OR 'tonsillitis'/exp OR 'tonsillar hyperplasia':ti,ab,kw | 21038 |
| #2 | 'tonsillectomy'/exp OR 'tonsillectomy' OR 'cold':ti,ab,kw OR 'radiofrequency':ti,ab,kw OR 'laser':ti,ab,kw OR 'ultrasound':ti,ab,kw OR 'microdebrider':ti,ab,kw OR 'surgery':ti,ab,kw | 34416 |
| #3 | 'randomized controlled trial'/exp OR 'randomized controlled trial' OR ('randomized controlled trial'/exp AND topic) OR 'randomized controlled trial':ti,ab,kw OR 'controlled study':ti,ab,kw OR 'clinical trial':ti,ab,kw | 1749238 |
| #4 | #1 AND #2 AND #3 | 432 |
| Cochran library |  |  |
| #1 | MeSH descriptor: [Tonsillitis] in all MeSH products | 515 |
| #2 | (Chronic Tonsillitis):ti,ab,kw OR (Tonsillitis, Chronic):ti,ab,kw | 343 |
| #3 | #1 OR #2 | 775 |
| #4 | MeSH descriptor: [Surgical Procedures, Operative] explode all trees | 177132 |
| #5 | (Surgical Technique*):ti,ab,kw OR (Surgical Procedure*):ti,ab,kw OR (Surgical Operation*):ti,ab,kw OR (Surgical Intervention*):ti,ab,kw OR (surgical Method*):ti,ab,kw | 134006 |
| #6 | #4 OR #5 | 260087 |
| #7 | MeSH descriptor: [Randomized Controlled Trials as Topic] explode all trees | 62740 |
| #8 | (Randomized Controlled Trial*):ti,ab,kw OR (RCT):ti,ab,kw OR (rct):ti,ab,kw OR (Randomized Trial*):ti,ab,kw OR (Controlled Trial*):ti,ab,kw | 1107551 |
| #9 | #7 OR #9 | 1107416 |
| #10 | #3 AND #6 AND #9 | 140 |

|  | Records identified from other sources | Exclusion Criteria |
| --- | --- | --- |
| 1 | Parsons SP, Cordes SR, Comer B. Comparison of posttonsillectomy pain using the ultrasonic scalpel, coblator, and electrocautery. Otolaryngol Head Neck Surg. 2006;134(1):106-113.  doi:10.1016/j.otohns.2005.09.027 | Control group did not undergo CD. |
| 2 | Parker D, Howe L, Unsworth V, Hilliam R. A randomised controlled trial to compare postoperative pain in children undergoing tonsillectomy using cold steel dissection with bipolar haemostasis versus coblation technique. *Clin Otolaryngol*. 2009;34(3):225-231.  doi:10.1111/j.1749-4486.2009.01932.x | Diagnosis was not uncomplicated chronic tonsillitis |
| 3 | Shapiro N L , Bhattacharyya N .Cold dissection versus coblation-assisted adenotonsillectomy in children.[J].Laryngoscope, 2010, 117(3).  DOI:10.1097/MLG.0b013e31802ffe47. | Target population underwent adenotonsillectomy |

# Supplement 2. Different categories data re-integrated for studies

| Categories | Study | Minimally invasive group | CD group |
| --- | --- | --- | --- |
| **Operative Time** | Raut 2001 | Median 13 (range, 3-55),100 | Median 20 (range, 6-50),100 |
|  | Raut 2001 re-integrat | Mean, SD, N  23.67 ± 11.33, 100 | Mean, SD, N  25.33 ± 9.59, 100 |
|  | Raut 2002 | Median 10.5 (range, 5-22),32 | Median 14.5 (range, 6-45),18 |
|  | Raut 2002 re-integrat | Mean, SD, N  12.50 ± 4.32, 32 | Mean, SD, N  21.83 ± 10.74, 18 |
|  | Elbadawey 2015 | Mean, SD, N  DL:15 ± 0.83, 40  Coblation:10 ± 0.99, 40 | 20 ± 1.0, 40 |
|  | Elbadawey 2015  re-integrat | 12.5 ± 2.67, 80 | 20 ± 1.0, 40 |
|  | Mahmut 2013 | Mean, SD, N  BCD: 17.73 ± 4.18, 39  TWS: 18.94 ± 3.87, 41 | 35.75 ± 10.15, 40 |
|  | Mahmut 2013 re-integrat | 18.36 ± 4.08, 80 | 35.75 ± 10.15, 40 |
|  | Kondra 2024 | Mean, SD, N  RF:17.83 ± 2.2, 12  BRF:16.30 ± 1.77, 12  CO2 Laser:16.4 ± 2.11, 10 | 18.87 ± 2.15, 24 |
|  | Kondra 2024  re-integrat | 16.89 ± 2.24, 34 | 18.87 ± 2.15, 24 |
|  | Hasan 2008 | Coblation:  Median 20.5 (range, 11-45),20 | BS  Median 12.0 (range, 6-19),20 |
|  | Hasan 2008 re-integrat | 24.25 ± 8.5, 20 | 12.25 ± 3.25, 20 |
| **Intraoperative Bleeding** | Raut 2001 | Median 5 (range, 0-391),100 | Median 115 (range, 16-642),100 |
|  | Raut 2001 re-integrat | Mean, SD, N  132.00 ± 85.19, 100 | Mean, SD, N  257.67 ± 136.38, 100 |
|  | Raut 2002 | Median 6 (range, 0-121),32 | Median 86 (range, 16-469),18 |
|  | Raut 2002 re-integrat | Mean, SD, N  42.33 ± 30.71, 32 | Mean, SD, N  190.33 ± 124.79, 18 |
|  | Elbadawey 2015 | Mean, SD, N  DL:25 ± 0.83, 40  Coblation:20 ± 0.85, 40 | 30 ± 1.0, 40 |
|  | Elbadawey 2015  re-integrat | 22.5 ± 2.65, 80 | 30 ± 1.0, 40 |
|  | Mahmut 2013 | Mean, SD, N  BCD: 9.88 ± 3.98, 39  TWS: 10.13 ± 4.14, 41 | 38.0 ± 10.83, 40 |
|  | Mahmut 2013 re-integrat | 10.01 ± 4.04, 80 | 38.0 ± 10.83, 40 |
|  | Kondra 2024 | Mean, SD, N  RF:30 ± 6.3, 12  BRF:16.25 ± 4.8, 12  CO2 Laser:12.8 ± 5.2, 10 | 105.4 ± 24.31, 24 |
|  | Kondra 2024  re-integrat | 20.09 ± 9.17, 34 | 105.4 ± 24.31, 24 |
| **Pain score** | Elbadawey 2015 | Mean, 95CI, N  D1:  DL:4.3, [4.16, 4.66], 40  Coblation:3.9, [3.68, 4.12], 40  D7  DL:1.7, [1.56, 1.84], 40  Coblation:1.5, [1.34, 1.66], 40 | Mean, 95CI, N  D1  4.5, [3.43, 4.66], 40  D7:  1.5,[1.34, 1.66], 40 |
|  | Elbadawey 2015  re-integrat | Mean, SD, N  D1  DL:4.3 ± 0.83,40 Coblation: 3.9 ± 0.71,40  D7  DL:1.7 ± 0.45, 40 Coblation: 1.5 ± 0.52, 40  D1 4.1 ± 0.78, 80  D7 1.6 ± 0.49, 80 | Mean, SD, N  D1:  4.5 ± 1.98, 40  D7:  1.5 ± 0.52, 40 |
|  | Mahmut 2013 | Mean, SD, N  BCD: 6.35 ± 1.2, 39 TWS: 4.7 ± 1.3, 41 | Mean, SD, N  4.5 ± 1.2, 40 |
|  | Mahmut 2013 re-integrat | Mean, SD, N  5.50 ± 1.2, 80 | 4.5 ± 1.2, 40 |
|  | Kondra 2024 | Mean, SD, N  24h:  RF:7.1 ± 0.8, 12  BRF:7.3 ± 0.6, 12  CO2 Laser:6.9 ± 0.7, 10  1w:  RF:4.5 ± 0.9, 12  BRF:4 ± 0.9, 12  CO2 Laser:3.1 ± 0.9, 10  2w:  RF:2.3 ± 0.4, 12  BRF:2.2 ± 0.9, 12  CO2 Laser:4.5 ± 0.9, 10  4w:  RF:0.16 ± 0.3, 12  BRF:0.33 ± 0.4, 12  CO2 Laser:0.5 ± 0.5, 10 | Mean, SD, N  24h:6.9 ± 0.7, 24  1w:3.1 ± 0.7, 24  2w:1.2 ± 0.5, 24  4w:0.08 ± 0.2, 24 |
|  | Kondra 2024  re-integrat | 24h: 7.11 ± 0.72, 34  1w:3.91 ± 1.46, 34  2w:2.91 ± 1.46, 34  4w:0.32 ± 0.42, 34 | 24h:6.9 ± 0.7, 24  1w:3.1 ± 0.7, 24  2w:1.2 ± 0.5, 24  4w:0.08 ± 0.2, 24 |

# Supplement 3：Sensitivity analysis, meta-regression and subgroup of operative time

**Forest plot of operative time exclude Raut 2001 and Raut 2002**

**Forest plot of operative time exclude Elbadawey 2015, Mahmut 2013 and Kondra 2024**

**Sensitivity analysis of operative time.**

**Forest plot of operative time exclude Kondra 2024**

**
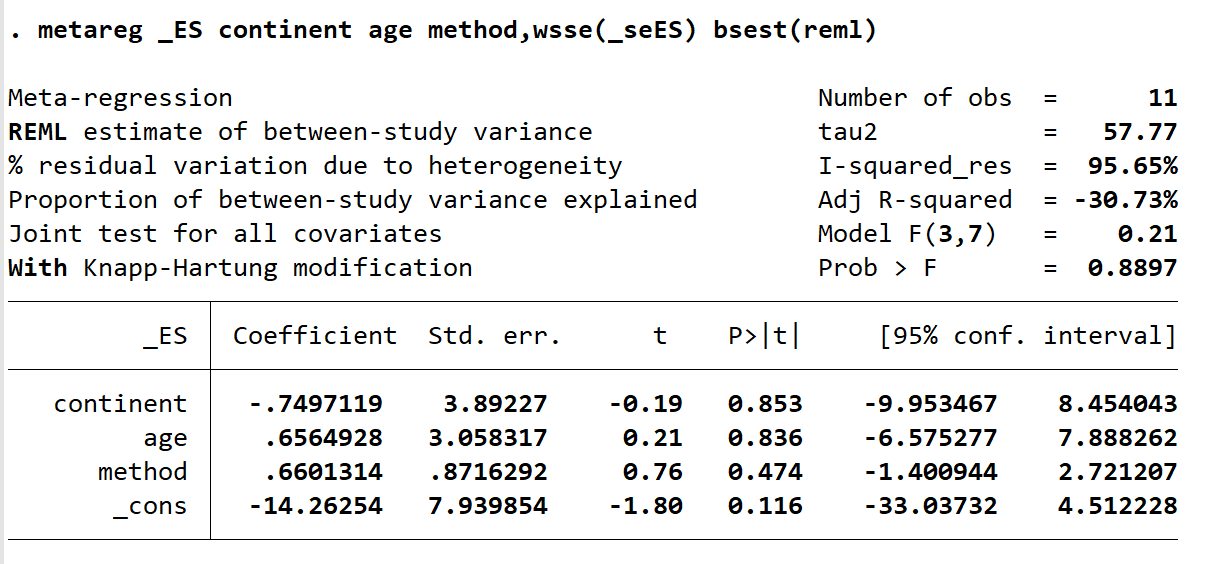
**

**Meta-regression of continent age and minimally invasive method**

NOTE 1=children 2=adults 3=mixed-age groups

**Forest plot of subgroup by age**

# Supplement 4：Sensitivity analysis, meta-regression and subgroup of Intraoperative Bleeding

**Forest plot of intraoperative bleeding exclude Raut 2001 and Raut 2002**

**Forest plot exclude Elbadawey 2015, Mahmut 2013 and Kondra 2024**

**Sensitivity analysis of intraoperative bleeding.**

**Forest plot exclude Ali 2020, Javid 2023 and Elbadawey 2015**


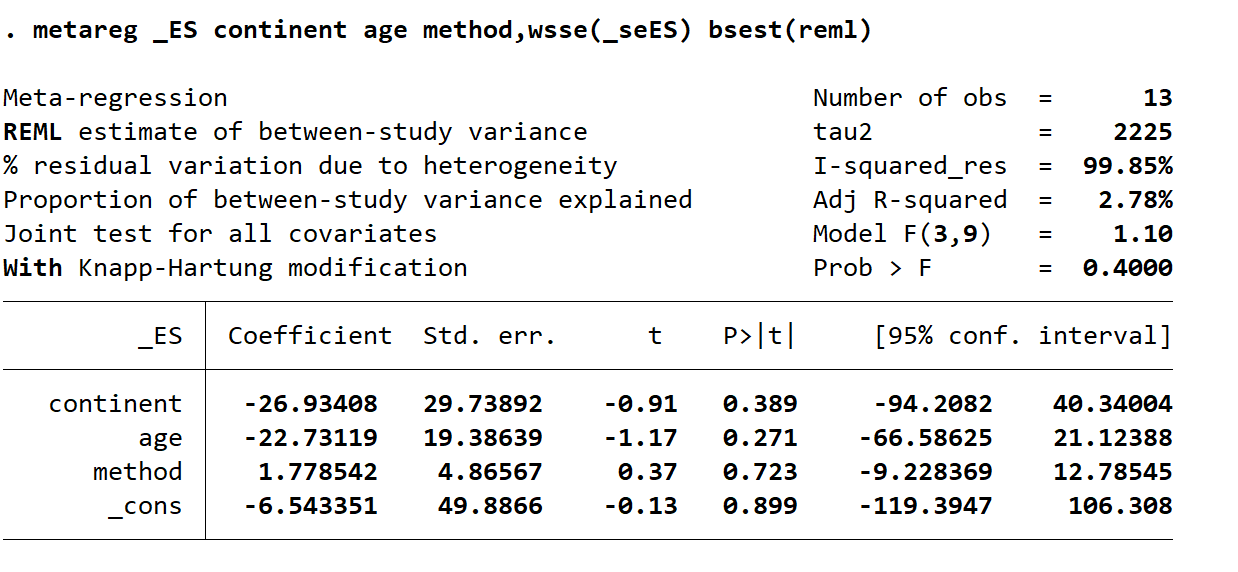


**Meta-regression of continent age and minimally invasive method**

NOTE 1=children 2=adults 3=mixed-age groups

**Forest plot of subgroup by age**

NOTE 1=Asia 2=Europe

**Forest plot of subgroup by continent**

# Supplement 5：Sensitivity analysis of VAS

**Forest plot of VAS exclude Kondra 2024**

# Supplement 6: Pain comparison outcomes

| **No.** | **study** | **evaluation indices** | ***P*** | **Study conclusion** | **Conclusion** |
| --- | --- | --- | --- | --- | --- |
| 1 | Raut 2001[19] | VAS | *P*＞0.05 | overall mean pain score was 6.9 for each group | No difference |
| 2 | Sugiura 2002[20] | VAS | *P*＞0.05 | UT group presented slightly higher mean VAS pain scores during the 6-day period (except for the second day). | **CD win** |
| 3 | Raut 2002 | VAS | *P*＞0.05 | The overall  pain score was 45.7 for the CD group and  43.1 for the BS group. | **Minimally Invasive (BS) win** |
| 4 | Oko 2005[22] | Face pain scale: | *P＜0.05* | BD(CD) group of pain scores lower than US group. | **CD win** |
| 5 | Philpott 2005[11] | VAS | *P*＞0.05 | No difference | No difference |
| 6 | Stavroulaki 2007[17] | VAS | D1-D4 *P＜0.05* | The duration to cessation of significant pain in the TWS group was shorter than that in the CD group. | **Minimally Invasive (TWS) win** |
|  |  |  | D5-D10 *P*＞0.05 |  | **Minimally Invasive (TWS) win** |
| 6 | Sezen 2008[23] | VAS | morning time pain scores  D1, D2  *P*＜0.01 | TWS shows lower than CD | morning time pain:  **Minimally Invasive (TWS) win** |
|  |  |  | morning time pain scores  D3-D7  *P*＞0.05 |  | **Minimally Invasive (TWS) win** |
|  |  |  | evening pain scores  D1-D7  *P*＞0.05 | TWS group shows lower evening pain scores in D1-D7 *P*＞0.05. | evening time pain:  **Minimally Invasive (TWS) win** |
| 7 | Guo 2012[12] | VAS | *P* <0.05 | duration of pain in the Coblation was shorter than CD | **Minimally Invasive (Coblation) win** |
| 8 | Mahmut 2013[25] | VAS | *P＞*0.05 | CD group shows lower pain scores than TWS | **CD win** |
|  |  |  | *P*＜0.05 | CD group shows lower pain scores than BCD | **CD win** |
|  |  |  | *P*＜0.05 | CD group shows lower pain scores than BCD | **CD win** |
| 9 | Celebi 2013[13] | Pain/discomfort score: | *P*＜0.05 | TWS group shows lower pain scores than CD | **Minimally Invasive (TWS) win** |
| 10 | Thangavel 2018[16] | VAS | D1,D7 *P*＜0.05 | CD group shows lower pain scores than CO2 | **CD win** |
| 11 | Ifthikhar 2018 | VAS | *P*＞0.05 | Laster group shows lower pain scores than CD | **Minimally Invasive (Laster) win** |
| 12 | Ali 2020[24] | FACES | *P*＜0.01 | RF group shows lower pain scores than CD | **Minimally Invasive (RF) win** |
| 13 | Elbadawey 2015[14] | FACES | D1:  DL vs CD  *P*＜0.05  DL vs Coblation *P*＜0.05  Coblation vs CD *P*＜0.05 | Coblation group shows lower pain scores than CD and DL. | Coblation win |
|  |  |  | D7:  DL vs CD  *P*＜0.05  DL vs Coblation *P*＜0.05 | Coblation and CD group shows lower pain scores than DL. | Coblation and CD win |
|  |  |  | D14: *P*＞0.05 | No difference | No difference |
| 14 | Kondra 2024[27] | VAS | 24h *P*＞0.05 | CO2 and CD was lower | **CO2 and CD win** |
|  |  |  | 1w *P*＜0.05 | CO2 and CD was lower | **CO2 and CD win** |
|  |  |  | 2w *P*＜0.05 | CD was lowest | **CD win** |
|  |  |  | 4w *P*＞0.05 | CD was lowest | **CD win** |

Note:

Dark green indicates pain in Minimally Invasive is lower than CD group, with P < 0.05.

Light green indicates pain in Minimally Invasive is lower than CD group, with P > 0.05.

Light red indicates pain in Minimally Invasive is higher than CD group, with P > 0.05.

Dark red indicates pain in Minimally Invasive is higher than CD group, with P < 0.05.

# Supplement 7: Hemorrhage outcomes

| **No.** | **study** | **Primary hemorrhage** | **Management** | **Secondary hemorrhage** | **Management** |
| --- | --- | --- | --- | --- | --- |
| 1 | Raut 2001[19] | 4 cases | conservative management. | BS:14/92  CD:17/92 | 18 received conservative treatment after readmission,  1 patient in CD group underwent hemostatic surgery again under general anesthesia,  6 were managed by general practitioners,  6 had minor bleeding but did not seek medical treatment. |
| 2 | Raut 2002[21] | BS:1/18  CD:1/32 | conservative management. | BS:3/18  CD:4/32 | 4 received conservative management,  3 had minor bleeding but did not seek medical treatment. |
| 3 | Oko 2005[22] | UT:1/61  CD:1/61 | NA | UT:8/61  CD:6/61 | 1 return to operative room. |
| 4 | Philpott 2005[11] | Coblation:0  CD:0 | - | Coblation:11/35  CD:8/36 | None:  Coblation:0 CD:2  GP visit:  Coblation:7 CD:3  Hospital visit:  Coblation:2 CD:1  Hospital admission  Coblation:2 CD:2 |
| 5 | Stavroulaki 2007[17] | TWS:0/25  CD:2/25 | ligation in operative room | TWS:0/25  CD:1/25 | D7 treated with silver nitrate cauterization. |
| 6 | Sezen 2008[23] | TWS:0  CD:0 | *-* | TWS:0  CD:0 | - |
| 7 | Guo 2012[12] | Coblation:0/25  CD:1/39 | NA | Coblation:2/25  CD:1/39 | NA |
| 8 | Mahmut 2013[25] | BCD:1/40  CD:1/40  TWS:0 | NA | BCD:2/40  CD:2/40  TWS:1/40 | NA |
| 9 | Ali 2020[24] | CD:1/97  RF:0/97 | surgical intervention | CD:1/97  RF:0/97 | conservative management. |
| 10 | Elbadawey 2015[14] | NA | NA | Coblation:1/40  CD:1/40  DL:0/40 | conservative management. |
| 11 | Kondra 2024[27] | RF:0  BRF:0  CO_2_:0  CD:0 | - | RF:1/12  BRF:0/12  CO_2_:0/10  CD:0/24 | conservative management. |

NOTE:NA=Not Mention

Dark green indicates that the incidence rate in the minimally invasive group is lower than that in the CD group.

Red indicates that the incidence rate in the CD group is higher than that in the minimally invasive group.

Yellow indicates that the incidence rate in the CD group is equal with the minimally invasive group.

White indicates we don’t know.

# Supplement 8: Publication bias of operative time

Funnel plot

Egger’s test


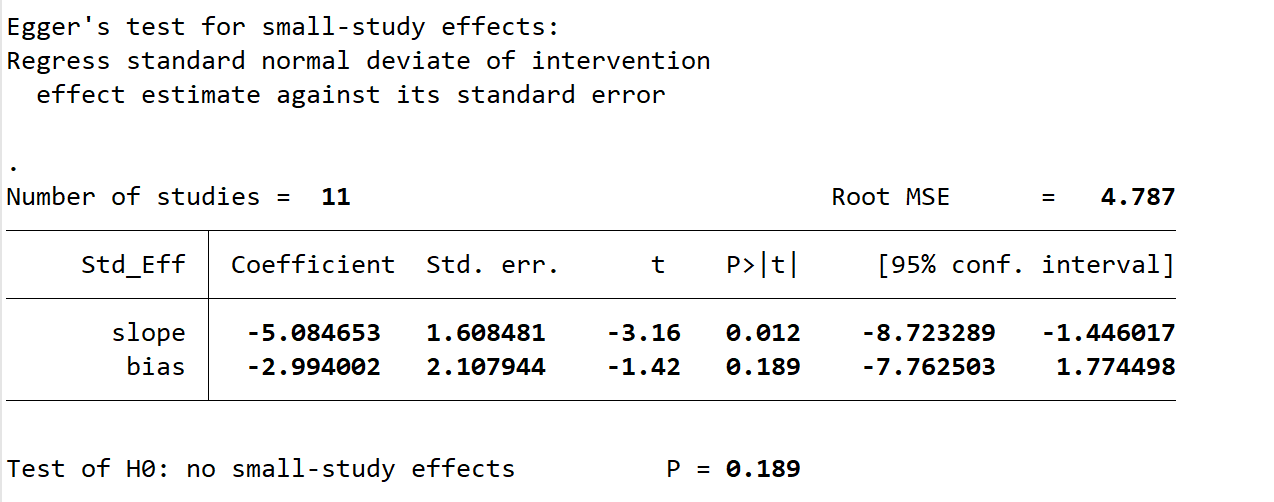


Trim-and-fill


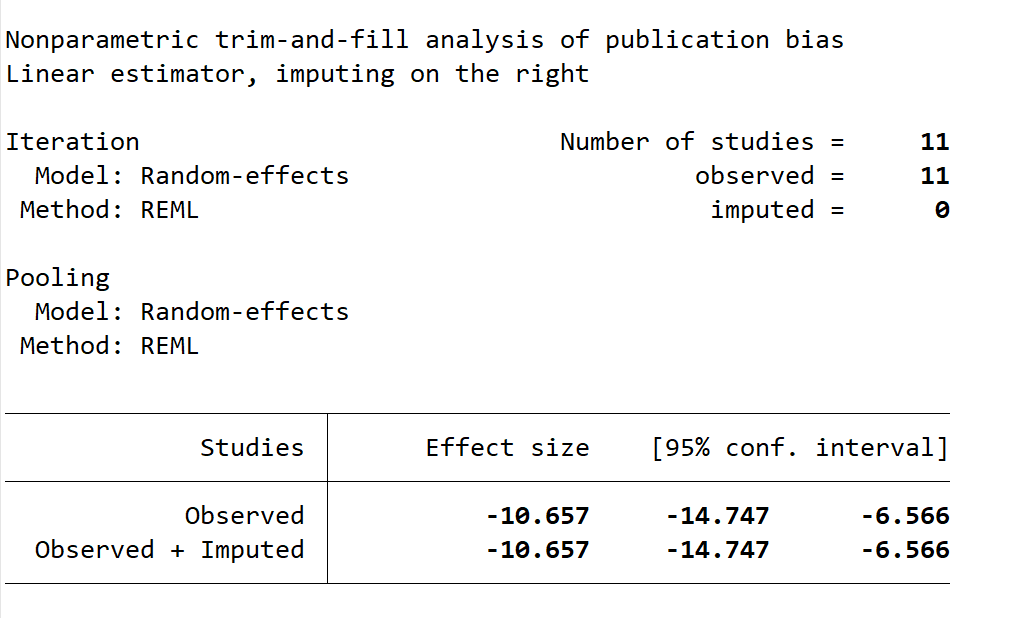


# Supplement 9: Publication bias of intraoperative bleeding

Funnel plot

#

Egger’s test


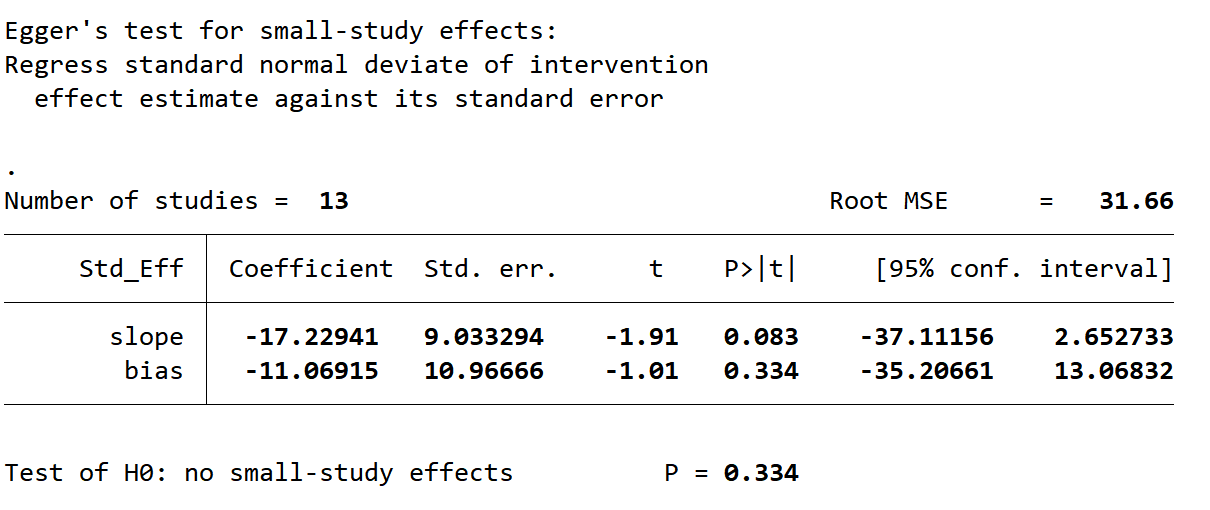


Trim-and-fill


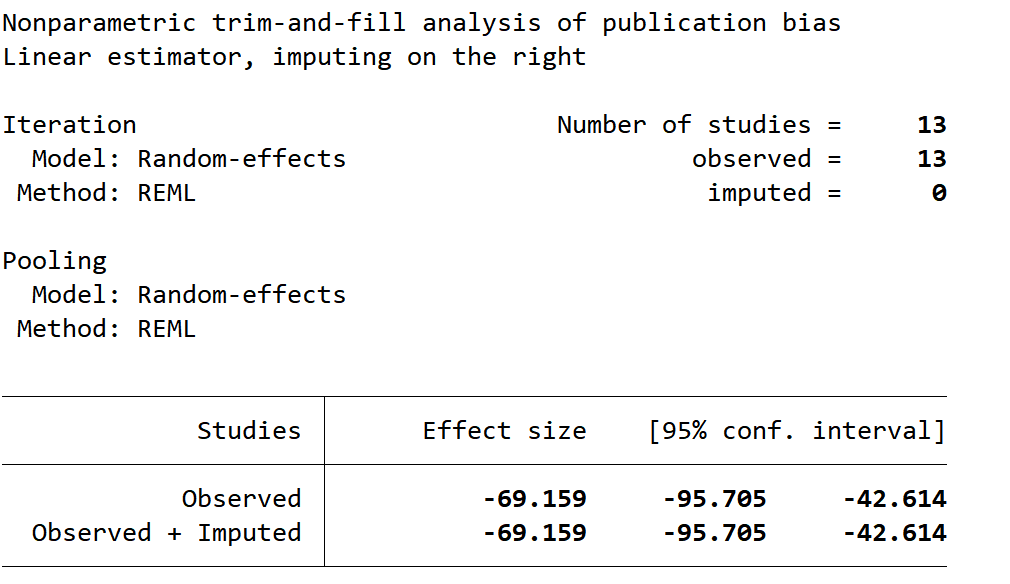


# Supplement 10: Grade evidence


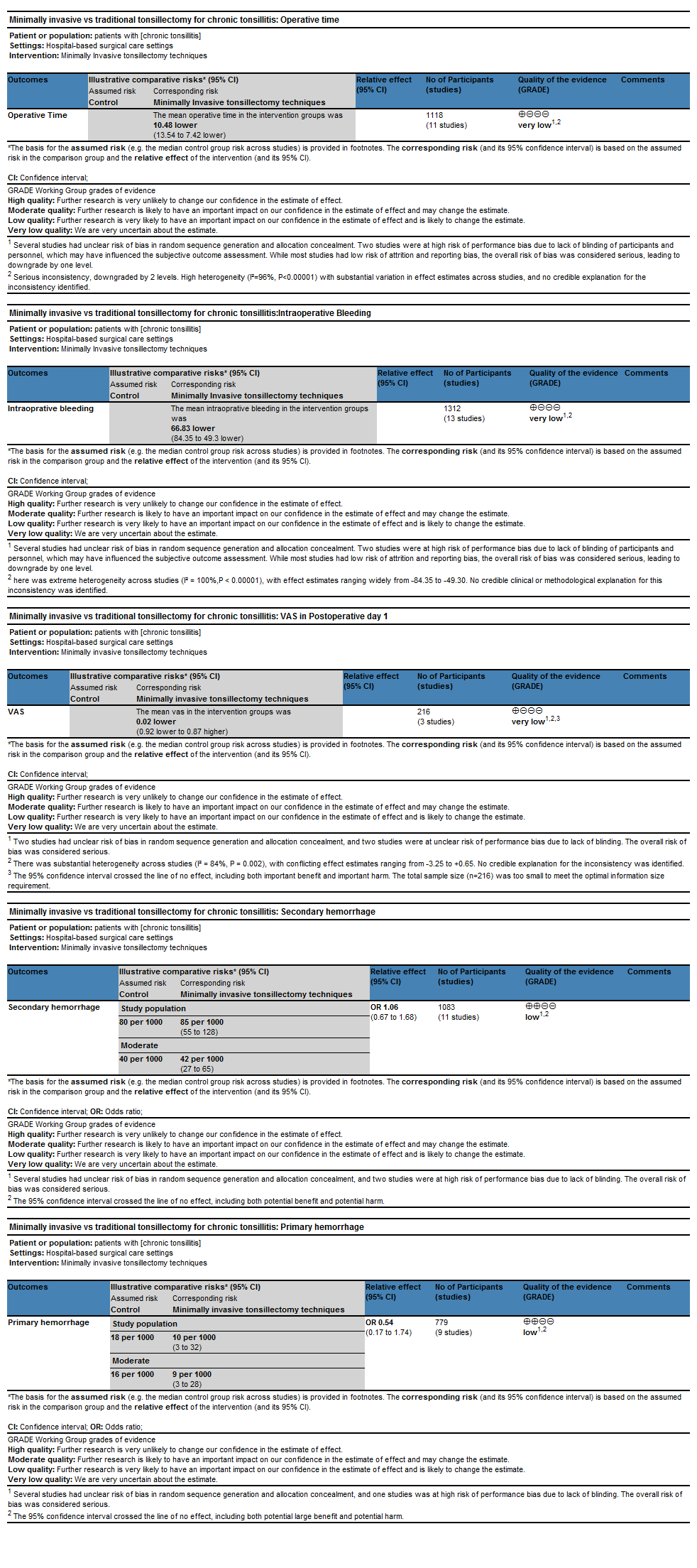

Supplement: Supplementary file 1 [file Datasheet1.docx]
